# Assessing the Impact of COVID-19 on Amateur Runners’ Performance: An Analysis through Monitoring Devices

**DOI:** 10.3390/s24082635

**Published:** 2024-04-20

**Authors:** María García-Arrabé, María-José Giménez, Juliette Moriceau, Amandine Fevre, Jean-Sebastien Roy, Ángel González-de-la-Flor, Marta de la Plaza San Frutos

**Affiliations:** 1 Faculty of Sport Sciences, Universidad Europea de Madrid, Tajo s/n, 28670 Villaviciosa de Odón, Spain; maria.gararrabe@universidadeuropea.es (M.G.-A.); juliette.moriceau@hotmail.fr (J.M.); amandinefevre24@gmail.com (A.F.); angel.gonzalez@universidadeuropea.es (Á.G.-d.-l.-F.); marta.delaplaza@universidadeuropea.es (M.d.l.P.S.F.); 2 Department of Rehabilitation, Faculty of Medicine, Université Laval, Quebec City, QC 2325, Canada; jean-sebastien.roy@fmed.ulaval.ca

**Keywords:** devices, real-world data, GPS, performance, COVID-19, amateur runners

## Abstract

This retrospective study aimed to analyze the return to running of non-professional runners after experiencing asymptomatic or mild COVID-19. Participants aged 18–55 years who maintained a training load of ≥10 km/week for at least three months prior to diagnosis and utilized Garmin/Polar apps were included. From these devices, parameters such as pace, distance, total running time, cadence, and heart rate were collected at three intervals: pre-COVID, immediately post-COVID, and three months after diagnosis. The Wilcoxon signed rank test was used for analysis (significance was set at ≤0.05). Twenty-one participants (57.1% male; mean age 35.0 ± 9.8 years) were included. The results revealed a significant decrease in running duration and distance two weeks after diagnosis, without significant changes in other parameters. Three months after infection, no differences were observed compared to pre-infection data, indicating a return to the pre-disease training load. These findings underscore the transient impact of COVID-19 on training performance among non-professional runners with mild or asymptomatic symptoms, highlighting the importance of tailored strategies for resuming running after infection.

## 1. Introduction

The COVID-19 pandemic has significantly affected physical activity in all age groups, and in most countries, with a greater negative effect on previously active individuals [1]. Social isolation during quarantines also reduced levels of physical activity, contributing to a loss in strength, cardiopulmonary capacity, and power [2,3]. Vaccination has been a crucial strategy in combating the pandemic, but with a changing acceptance in the population over time [4]. Although many patients recover from the disease without sequelae, the coronavirus can cause respiratory problems, fever, and lung inflammation, especially in vulnerable individuals [5]. Approximately 10% to 20% of patients who have had COVID-19, even without symptoms, experience lingering effects beyond 12 weeks after diagnosis [6]. This includes possible impacts on physical activity. A previous study reported that patients with severe forms of COVID-19 (hospitalized recovered women) resumed physical exercise but not at the same intensity as before the infection [7]. Specifically, running time for non-COVID-19-affected runners was 56% longer than that of runners who had contracted COVID-19, and their speed was 26% higher [7].

Recent research has revealed that the impact of COVID-19 transcends cardiopulmonary functions, also affecting the musculoskeletal system and running biomechanics [8]. Among the primary post-infection symptoms is the persistence of fatigue, suggesting a possible long-term impact on body energy systems and muscle recovery processes [9].

Variations in running kinetics and electromyography have been observed in runners with a history of COVID-19, supporting the notion that the effects of the virus are not merely temporary and are not limited to the period of acute infection [2,10]. Furthermore, they suggest that they may lead to persistent alterations in muscle activation patterns and running biomechanics, thereby increasing the risk of injury in runners who have suffered from the disease.

GPS (global positioning system) has evolved from its military origin in the 1960s to become a key tool in disease monitoring and management [11]. In the contemporary era, the combination of GPS devices with other technologies has significantly transformed the landscape of sports and health sciences, opening up new possibilities in this field, such as recording and tracking physical activity, heart rate monitoring, location tracking, and symptom tracking [12]. Leading brands such as Garmin^®^ and Polar^®^ are at the forefront of this technological revolution, offering a wide range of products capable of objectively measuring a multitude of running parameters [13]. Previous studies have utilized these devices, which are validated for accuracy and reliability [14].

Understanding the full extent of the impact of COVID-19 on runners requires a comprehensive approach that takes into account both immediate and long-term effects. Wearable devices provide valuable information by accurately recording parameters such as pace, distance, time, and even complex physiological data such as maximum and average heart rate [15]. The integration of these data can be beneficial in understanding training dynamics, as well as fundamental tools in health and sports science research, facilitating a deeper understanding of the physiological and biomechanical aspects of running.

While significant attention has understandably been directed toward the impact of severe or hospitalized cases of COVID-19 on athletes, there is a notable lack of research concerning the short-, medium-, and long-term effects of asymptomatic or mild COVID-19 infections among active individuals such as runners. Gaining insight into the impact of asymptomatic/mild infections within this demographic group is crucial, as it could affect their training performance and overall wellbeing.

This retrospective observational study aimed to determine the effect of asymptomatic or mild COVID-19 on the return to running immediately and three months post-infection using metrics provided by GPS devices (Polar^®^, Kempele, Finland; Garmin^®^, Olathe, KS, USA).

## 2. Materials and Methods

### 2.1. Study Design

A retrospective, analytical, observational study was conducted through the examination of real-world data, capitalizing on the capabilities of GPS-enabled watches to document running parameters accurately.

### 2.2. Participants

Healthy non-professional runners between 18 and 55 years old were invited to participate if they complied with the following criteria: body mass index (BMI) of 20–25 kg/m^2^ and regular running training for at least 3 months prior to a confirmed diagnosis of COVID-19, with a weekly training distance of at least 10 km. Participants were also required to have their training data recorded in either the Garmin or Polar Flow applications, ensuring the availability of verifiable training data. A confirmed positive diagnosis of COVID-19 through either PCR or antigen testing was also a prerequisite.

Exclusion criteria were defined to omit participants with severe COVID-19 manifestations, characterized by symptomatic infection with respiratory distress, as evidenced by tachypnea and/or dyspnea and abnormal findings on chest X-ray (corresponding to Stages 1 and 2 of the Classification of Severity by the National Institute of Health Infection). Additionally, individuals with a history of cardiorespiratory diseases, neurodegenerative conditions, acute illnesses leading to hospitalization within the last six months, pregnancy, or cancer within the preceding five years were precluded from participation.

**Figure 1:**
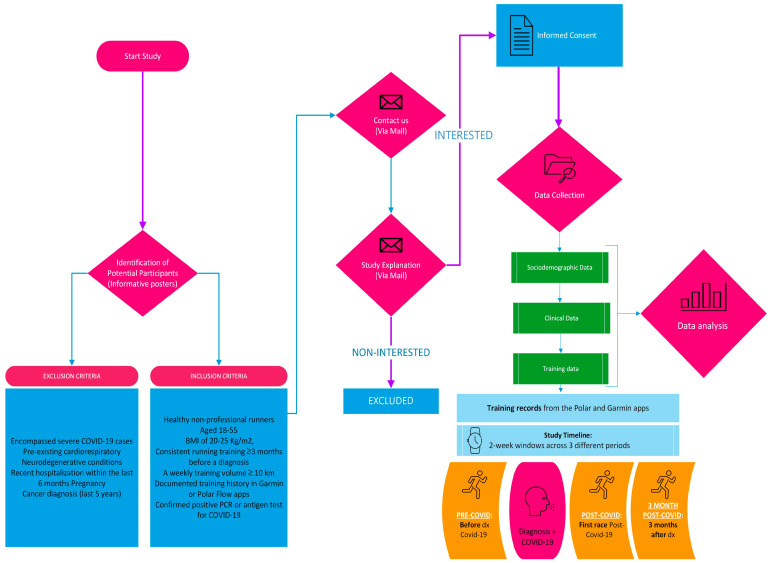
Flowchart detailing the recruitment and study processes.

Participant recruitment was executed through convenience sampling, utilizing informative posters strategically placed at the Faculty of Sport Sciences of Universidad Europea, Madrid, Spain. The recruitment and study methodology are comprehensively delineated in a flowchart (Figure 1), which elucidates the sequential processes involved in participant selection and data collection.

### 2.3. Procedures

In adherence with the ethical guidelines and legal requirements stipulated for biomedical research, this study meticulously followed the principles laid out in the Declaration of Helsinki, as well as the pertinent regulations in Spanish legislation concerning biomedical research, the protection of personal data, and digital rights. The Ethics Commission of Universidad Europea de Madrid (CIPI/23.051) granted approval for the study protocol, underscoring the research’s compliance with ethical standards and legal mandates. Before commencing data collection, eligible participants were provided with an informed consent form. This document thoroughly explained the objectives, procedures, and ethical considerations of the study, ensuring that participants were fully informed of the nature and implications of their involvement.

This study harnessed the technological capabilities of Garmin^®^ and Polar^®^ applications to collect detailed running and physiological data. Additionally, sociodemographic information (including age, sex, and country), weight, height, and smoking habits were recorded. Participants were also required to provide specific details regarding their acute COVID-19 infection, such as the date of diagnosis, vaccination details (type of vaccine and number of doses received prior to diagnosis), and the number of previous episodes before the acute episode under study.

**Figure 2:**
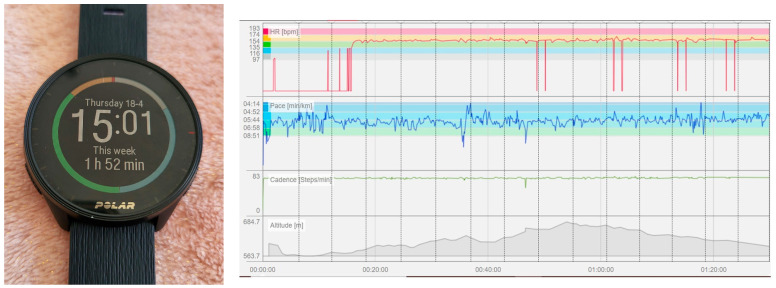
A fitness tracker watch and an example of variables collected through these wearable devices.

Figure 2 shows an image of a fitness tracker watch and an example of running records obtained from the application. From the Garmin or Polar flow applications, a comprehensive set of variables was extracted, including maximum pace (in minutes (min)/kilometer (km)), average pace (in min/km), total run distance (in km), total running time (in min), average and maximum cadence (steps/min), as well as maximum and average heart rate (beats/min).

**Maximum Pace (min/km):** This variable indicates the fastest speed at which the participant ran during a specific period, calculated as the minimum amount of time taken to cover one kilometer. It reflects the runner’s peak performance capability over the monitoring periods.

**Average Pace (min/km):** Unlike maximum pace, the average pace calculates the mean speed across all runs within the specified period, offering a broader overview of the participant’s general running speed.

**Total Distance Run (Km):** This quantifies the cumulative distance covered by the participant in all runs during each monitoring period, measured in Km. It serves as a metric of overall running activity and endurance.

**Total Running Time (min):** The sum of time spent running across all sessions within each period, measured in min. This variable reflects the total exercise duration and is essential for understanding overall training volume.

**Average Cadence (steps per min):** This is the average number of steps the participant took per minute across all runs in a period. Cadence is a crucial factor in running efficiency and injury prevention.

**Maximum Cadence (steps per min):** Reflects the highest steps per minute rate achieved by the participant during any run in the period. It indicates the peak leg turnover rate, which can be critical for sprinting or high-intensity intervals.

**Maximum Heart Rate (beats per min):** The highest heart rate recorded during the most intense physical exertion in a period. It is a vital indicator of cardiovascular exertion and the physical limits of the participant.

**Average Heart Rate (beats per min):** Calculates the mean heart rate across all running sessions within each period. This metric provides insight into the cardiovascular demand of the participant’s average running session and overall fitness level.

Data for these variables were collected for three distinct periods:

1. The two-week period prior to the diagnosis of the COVID-19 infection (PRE-COVID).
2. The two-week period following the first run post-COVID-19 infection (POST-COVID).
3. The two-week period three months after the diagnosis of the infection (3M-POSTCOVID).

**Figure 3:**
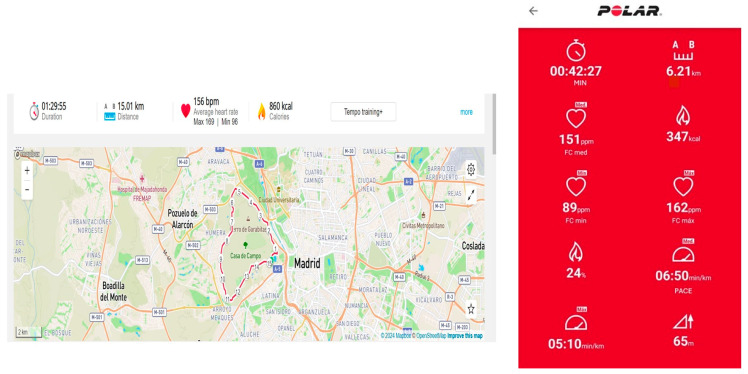
Example of information on running parameters from wearable devices.

Participants independently accessed their app records for the specified dates and submitted the data via screenshots to an email address designated for this purpose. Figure 3 shows an example of information on running parameters from wearable devices representing the screenshots received from participants.

Researchers responsible for data collection accessed these emails and entered the data into a specially designed database for the study. In line with ethical guidelines and legal requirements for the protection of personal data, all collected data were anonymized. Personally identifiable information was deliberately omitted from the database to safeguard participant privacy and confidentiality.

### 2.4. Statistical Analyses

The GRANMO application v.7 (https://www.imim.es/ofertadeserveis/software-public/granmo/; accessed on 10 October 2022) was used for sample size calculation. The number of participants was determined based on the variability in the mean run distance during the two-week period before the diagnosis of the COVID-19 infection in a pilot study including 10 participants. The mean (±SD) distance covered during that period was 11.85 ± 2.36 km. Differences of 15% were considered significant based on a previous study [16]. Considering a two-sided contrast with an alpha risk of 0.05 and a beta risk of 0.2, a total of 20 participants was required. This sample size would allow the detection of a difference equal to or greater than 1.5 km, assuming no participant drop-outs between recruitment and follow-up and considering a standard deviation of 2.36.

SPSS 25.0 software (IBM SPSS Statistics, IBM, Armonk, NY, USA) was used for data analysis. Mean and standard deviation or median and interquartile range were used for the description of quantitative variables, whereas for qualitative variables, the number and percentage of participants were used. The Mann–Whitney U test compared the post-COVID distance and duration of the group of runners who returned to running within 7 days of the diagnosis with the data of the group who returned to running more than 7 days after the diagnosis. The non-parametric Wilcoxon signed rank test was used for the following comparisons of pairwise data: PRE-COVID vs. POST-COVID; PRE-COVID vs. 3M-POST-COVID; and POST-COVID vs. 3M-POST-COVID. The level of significance was set at ≤0.05.

## 3. Results

A total of 21 participants (12 men, 57.1%) were included in the study; 16 (76.2%) of them were from France, three (14.29%) were from Colombia, and the remaining two (9.52%) were from Spain. Participants were recruited and data were collected during March and April 2023.

Table 1 shows demographic characteristics of the study participants. None of the participants were smokers.

COVID-19 infections in the participants occurred from 1 September 2020 to 25 December 2022; 3 (14.29%) occurred in 2020, 5 (23.81%) occurred in 2021, and 13 (61.9%) occurred in 2022. The COVID-19 episode from which data were collected was the first episode of infection for 15 (71.4%) participants, the second for 5 participants (23.8%), and the third one for 1 (4.8%) subject. In only 4 (19.1%) participants, the infection was asymptomatic. In the acute phase of the infection, among symptomatic participants, fatigue (82.4% participants) was the most frequent symptom followed by fever (76.5%), myalgia (64.7%), and arthralgia (11.8%). None of the participants exhibited post-acute sequelae of SARS-CoV-2 infection.

Table 1 also shows vaccination data from study participants. Among those vaccinated, the Pfizer-BioNTech COVID-19 vaccine had been administered to 13 (65.0%) participants, Pfizer-BioNTech followed by Moderna to 3 (15.0%), the Oxford/AstraZeneca followed by the Pfizer-BioNTech vaccine to 2 (10.0%), and Oxford/AstraZeneca and Sinovac to 1 (5.0%).

In the different periods, the number of runs from which the mean of the data was calculated was 5.86 ± 3.53 (range 2–13) in the PRE-COVID period, 6.24 ± 3.40 (range 2–12) in the POST-COVID period, and 6.67 ± 4.95 (range 1–19) in the 3M-POST-COVID period. The mean (IQR) time to return to running after the diagnosis was 5 (4, 10) days.

Thirteen participants (61.9%) returned to running during the first 7 days post-COVID-19 diagnosis, with a mean running time of 57.6 (±18.0) minutes and an average distance of 9.54 (±1.66) km. Eight participants (39.1%) that started after one week had a mean running time of 55.8 (±20.4) minutes and an average distance of 9.97 (±2.82) km. No significant differences (*p* = 0.562) were found between these two groups regarding running time and distance covered.

Table 2 shows running data for the three periods. Running in the POST-COVID period was shorter in time and distance, although differences were only statistically significant when comparing the PRE-COVID vs. POST-COVID period (*p* < 0.05). No statistically significant differences were found for the other parameters measured in the paired statistical analysis performed.

## 4. Discussion

Our findings, based on data extracted from Garmin^®^ or Polar^®^ fitness tracker watches, showed a significant decrease in running duration and distance during the two-week period after the first race following the COVID-19 episode. However, there were no significant changes in heart rate, pace, or cadence. The results obtained at 3 months after diagnosis indicated that the runners returned to similar training levels as before the COVID-19 infection.

Notably, none of the participants were smokers, a characteristic that significantly reduces the confounding impact of smoking on the study’s outcomes. This absence of smokers aligns with the study’s aim to examine variables potentially unaffected by the adverse health impacts of tobacco use, thus ensuring a cleaner analysis of the data.

Although the results did not support our hypothesis, the information has important clinical relevance for the sports community and health and sport professionals. The fact that COVID-19 infection did not appear to have a lasting negative impact on athletic performance and training ability provides a reassuring perspective for runners and their coaches. This information may help to reduce the anxiety and fear associated with returning to physical activity after recovery from illness. However, the individual variability in response to the disease is a characteristic of COVID-19 that should be considered.

In the absence of changes in objective parameters such as heart rate, a possible explanation for the decrease in the running variables measured during the first two weeks after the return to training could be attributed to the runner’s deliberate choice to begin with a reduced volume and intensity with respect to their pre-COVID-19 level after the training interruption.

Contrary to our results, evidence suggests that 10–20% of patients continue to experience disease effects beyond 12 weeks post-diagnosis [6]. COVID-19 has had significant effects on athletes, both on their physical health and athletic performance [17]. Some of the most commonly reported effects include respiratory symptoms [2], fatigue, weakness, cardiac complications [3], decreased strength, and power [8]. It is important to note that the effects of COVID-19 on athletes can vary considerably from one person to another. Some athletes may recover completely without experiencing lasting effects, while others may face health and athletic performance challenges, as noted in the study by Jafarnezhadgero et al. [7]. In that study, COVID-19-free runners completed 56% longer running times and 26% faster speeds, and the runners with COVID-19 showed compromised endurance and altered kinetics in the form of longer stance periods and weaker propulsive forces [7]. It is important to consider that all the female runners included in the experimental group had been hospitalized for COVID-19 at least 14 days before the study [7]. This condition may have significantly influenced their physical capabilities and recovery. The difference in findings with respect to our study can be attributed to the fact that our sample included runners who experienced no or mild symptoms of COVID-19 and did not require hospitalization. Therefore, it seems essential to consider the severity of the disease when interpreting these findings and comparing them to our own results.

By analyzing running parameters, it could be interpreted that runners in the present study were amateur runners with moderate experience. The duration and distance values of the workouts, with an average duration of around 60–70 min and an average distance of ~10 km, indicate a reasonable dedication to training but not at a professional level. They showed a relatively high average cadence (~165.76 steps per minute), suggesting a good running technique and possibly improved efficiency compared to less experienced runners. However, they did not reach the 180 steps per minute typical of high-performance athletes with excellent running technique, although they could increase their cadence considerably, reaching very efficient values of up to 193 steps per minute. The average and maximum heart rate were within moderate and safe ranges, suggesting sustained but not extremely intense efforts. Their running style was balanced; although there was a reduction in duration and distance POST-COVID compared to PRE-COVID, their performance did not seem to be significantly reduced. This suggests that runners maintained a healthy balance between effort and recovery, which might be indicative of a good understanding of training management.

Runners recovered their training load after 3 months, with non-significant differences in distance, duration, cadence, paces, average heart rate and maximum heart rate. In line with our results, Emeran et al. [17] analyzed the impact of COVID-19 on the training of runners and cyclists also using data through a GPS monitoring device. Their participants were compared to a control group of non-infected athletes whose training had been interrupted for between two to four weeks for different reasons. One week after training interruption, decreases in maximum and average heart rate, relative exercise intensity, maximum and average speed, time, and distance trained were observed in both the COVID-19 group and the control group, thus eliminating the possibility of a COVID-19-specific effect on training activity after infection. COVID-19 affected infected runners to the same extent that the training interruption affected the control group [17].

The results show that 95.23% of the runners who participated in the present investigation had been vaccinated at the time of contracting SARS-CoV-2 infection. Vaccination against COVID-19 has been widely studied for its efficacy in preventing serious illness, hospitalizations and deaths related to the virus [18], but specific studies directly linking physical fitness to vaccination against COVID-19 are very limited. Nevertheless, published articles have pointed out that regular and moderate physical exercise can have a positive impact on the immune system [16,19,20], positively contributing to protection against COVID-19 infection. It is noteworthy that participants in our study were young, trained, non-smokers, and most of them were vaccinated, a situation that may have influenced the recovery of the variables studied.

Several published studies follow the performance of athletes in controlled settings after the diagnosis of COVID-19, such as the study by Brito et al. [21] or Komici et al. [22]. Specifically, Brito et al. conducted a comparative study between athletes with persistent COVID symptoms and those without persistent symptoms. The results did not show statistically significant differences between both groups in ergospirometric parameters. Another study by Komici et al. [22], which also included the exercise test as a variable, demonstrated ventilatory inefficiency in participants, but it did not modify exercise capacity, aligning with our findings.

This study differs from previous research by not opting for traditional stress testing and instead focusing on the implementation of GPS systems, such as Polar^®^ and Garmin^®^. These devices, widely used by runners, allow for accurate and continuous real-time data collection during runs in uncontrolled environments, such as urban environments or rural roads, opting for a practical approach connected to the runners’ reality. This approach has the potential to be extrapolated to large-scale big data studies, similar to the approach proposed by Alsunaidi et al. [23]. Our goal was to provide valuable data that could contribute to the analysis of the complexity of performance following COVID-19 infection, thus providing a broader and more applicable perspective to understanding the short- and long-term effects of mild respiratory infections on physical activity.

### Limitations

Several limitations should be acknowledged. A primary limitation stems from the study’s reliance on the secondary analysis of recorded data. This approach inherently restricts the scope of available information, such as specific COVID-19 variants, characteristics of the terrain, and the interval between vaccination and infection. The absence of these critical data points potentially constrains the depth and applicability of our analysis. The study’s dependence on participants’ self-reported data, particularly regarding symptom severity and vaccination history, introduces an inherent susceptibility to bias. Despite efforts to ensure accurate and detailed information collection, the subjective nature of self-reporting cannot be entirely mitigated. This limitation could affect the reliability of the findings, especially when evaluating the severity of symptoms and their impact on running performance. The results, while providing valuable insights into the effects of COVID-19 on amateur runners, cannot be directly generalized due to the unknown and heterogeneous clinical presentations of COVID-19. The study’s sample, predominantly composed of young, trained, non-smoker subjects who were mostly vaccinated, may not represent the broader population of amateur runners, particularly those with different health backgrounds or those who have experienced severe COVID-19 symptoms.

## 5. Conclusions

This study, which examined real-world data from wearable devices, investigated how asymptomatic or mild COVID-19 infection affected the training performance of non-professional runners. The results showed that the return to training occurred early, at a median of 5 days after diagnosis. In the immediate onset, there was a marked decline in the athletes’ training performance, as evidenced by a significant decrease in both the run distance and the duration of their practice. Three months after the diagnosis of the infection, the athletes returned to their pre-infection training load. These results underline the adaptive capacity of young, trained, amateur runners to overcome limitations resulting from infection. This information may be valuable for the development of comprehensive and personalized strategies for the resumption of running after a mild respiratory infection. Future studies also integrating wearable technology and gathering more detailed data on terrain characteristics, specific COVID-19 variants, and precise timelines from vaccination to infection would be desirable. Additionally, the inclusion of a highly diverse cohort of runners will improve the generalizability of the findings, providing a more definitive understanding of the impact of mild viral infections on athletes.

## Figures and Tables

**Table 1:** Characteristics of the total sample.

Variables	
Age (years)	35.0 ± 9.8
Gender [males; n (%)]	12 (57.14)
Height (cm)	173.9 ± 8.5
Weight (kg)	66.1 ± 9.2
Vaccination; n (%)	20 (95.24)
No. of doses; n (% total sample):	
One	4 (19.05)
Two	8 (38.10)
Three	7 (33.33)
Four	1 (4.76)

Data are shown as mean ± SD except where indicated.

**Table 2:** Running variables at the three periods of data.

Variable	PRE-COVID	POST-COVID	3M-POST-COVID	*p* PRE-COVID vs. POST-COVID ^a^	*p* PRE-COVID vs. 3M-POST-COVID ^a^	*p* POST-COVID vs. 3M-POST-COVID ^a^
**Duration (min)**	64.29 ± 14.38	57.03 ± 18.46	67.08 ± 35.52	0.046	0.566	0.414
**Distance (km)**	10.93 ± 2.26	9.70 ± 2.12	10.93 ± 4.30	0.030	0.689	0.357
**Mean heart rate (bpm)**	151.98 ± 7.32	154.75 ± 9.46	152.10 ± 7.87	0.063	0.768	0.217
**Maximum heart rate (bpm)**	174.97 ± 9.83	175.0 ± 10.26	174.81 ± 9.37	0.794	0.664	0.434
**Mean pace (min/km)**	5.93 ± 0.68	5.89 ± 1.01	6.09 ± 0.94	0.578	0.230	0.191
**Maximum pace (min/km)**	4.87 ± 0.59	5.02 ± 0.62	5.14 ± 0.74	0.313	0.083	0.332
**Mean cadence (spm)**	165.76 ± 6.63	165.32 ± 7.56	164.67 ± 9.44	0.614	0.498	0.781
**Maximum cadence (spm)**	193.38 ± 17.58	191.10 ± 15.27	194.91 ± 21.35	0.434	0.903	0.498

Data are expressed as mean ± SD; ^a^ Wilcoxon signed rank test; bpm: beats per minute; spm: steps per minute.

## Data Availability

Datasets supporting the reported results are available upon request to the first author.

## References

[1] Wunsch K., Kienberger K., Niessner C. (2022). Changes in Physical Activity Patterns Due to the COVID-19 Pandemic: A Systematic Review and Meta-Analysis. Int. J. Environ. Res. Public Health.

[2] Damiot A., Pinto A.J., Turner J.E., Gualano B. (2020). Immunological implications of physical inactivity among older adults during the COVID-19 pandemic. Gerontology.

[3] López-Bueno R., Calatayud J., Andersen L.L., Casaña J., Ezzatvar Y., Casajús J.A., López-Sánchez G.F., Smith L. (2021). Cardiorespiratory fitness in adolescents before and after the COVID-19 confinement: A prospective cohort study. Eur. J. Pediatr..

[4] Norhayati M.N., Che Yusof R., Azman Y.M. (2022). Systematic Review and Meta-Analysis of COVID-19 Vaccination Acceptance. Front. Med. Lausanne.

[5] Atzrodt C.L., Maknojia I., McCarthy R.D.P., Oldfield T.M., Po J., Ta K.T.L., Stepp H.E., Clements T.P. (2020). A guide to COVID-19: A global pandemic caused by the novel coronavirus SARS-CoV-2. FEBS J..

[6] Nalbandian A., Sehgal K., Gupta A., Madhavan M.V., McGroder C., Stevens J.S., Cook J.R., Nordvig A.S., Shalev D., Sehrawat T.S. (2021). Post-acute COVID-19 syndrome. Nat. Med..

[7] Jafarnezhadgero A.A., Noroozi R., Fakhri E., Granacher U., Oliveira A.S. (2022). The impact of COVID-19 and muscle fatigue on cardiorespiratory fitness and running kinetics in female recreational runners. Front. Physiol..

[8] Lopez-Leon S., Wegman-Ostrosky T., Perelman C., Sepulveda R., Rebolledo P.A., Cuapio A., Villapol S. (2021). More than 50 long-term effects of COVID-19: A systematic review and meta-analysis. Sci. Rep..

[9] Tuzun S., Keles A., Okutan D., Yildiran T., Palamar D. (2021). Assessment of musculoskeletal pain, fatigue and grip strength in hospitalized patients with COVID-19. Eur. J. Phys. Rehabil. Med..

[10] Jafarnezhadgero A.A., Hamlabadi M.P., Sajedi H., Granacher U. (2022). Recreational runners who recovered from COVID-19 show different running kinetics and muscle activities compared with healthy controls. Gait Posture.

[11] Sun X., Wang Z., Fu X., Zhao C., Wang F., He H. (2023). Validity of Apple Watch 6 and Polar A370 for monitoring energy expenditure while resting or performing light to vigorous physical activity. J. Sci. Med. Sport.

[12] Jerath R., Syam M., Ahmed S. (2023). The Future of Stress Management: Integration of Smartwatches and HRV Technology. Sensors.

[13] Henriksen A., Haugen Mikalsen M., Woldaregay A.Z., Muzny M., Hartvigsen G., Hopstock L.A., Grimsgaard S. (2018). Using Fitness Trackers and Smartwatches to Measure Physical Activity in Research: Analysis of Consumer Wrist-Worn Wearables. J. Med. Internet Res..

[14] Kaewkannate K., Kim S. (2016). A comparison of wearable fitness devices. BMC Public Health.

[15] Sirisunhirun P., Bandidniyamanon W., Jrerattakon Y., Muangsomboon K., Pramyothin P., Nimanong S., Tanwandee T., Charatcharoenwitthaya P., Chainuvati S., Chotiyaputta W. (2022). Effect of a 12-week home-based exercise training program on aerobic capacity, muscle mass, liver and spleen stiffness, and quality of life in cirrhotic patients: A randomized controlled clinical trial. BMC Gastroenterol..

[16] Edwards K.M., Booy R. (2013). Effects of exercise on vaccine-induced immune responses. Hum. Vaccin. Immunother..

[17] Emeran A., Lambert E.V., Paruk T., Bosch A. (2022). Changes in training activity post COVID-19 infection in recreational runners and cyclists. S. Afr. J. Sports Med..

[18] Graña C., Ghosn L., Evrenoglou T., Jarde A., Minozzi S., Bergman H., Buckley B.S., Probyn K., Villanueva G., Henschke N. (2022). Efficacy and safety of COVID-19 vaccines. Cochrane Database Syst. Rev..

[19] Chastin S.F.M., Abaraogu U., Bourgois J.G., Dall P.M., Darnborough J., Duncan E., Dumortier J., Pavón D.J., McParland J., Roberts N.J. (2021). Effects of regular physical activity on the immune system, vaccination and risk of community-acquired infectious disease in the general population: Systematic review and meta-analysis. Sports Med..

[20] Dixit S. (2020). Can moderate intensity aerobic exercise be an effective and valuable therapy in preventing and controlling the pandemic of COVID-19?. Med. Hypotheses.

[21] Brito G.M., do Prado D.M.L., Rezende D.A., de Matos L.D.N.J., Loturco I., Vieira M.L.C., de Sá Pinto A.L., Alô R.O.B., de Albuquerque L.C.A., Bianchini F.R. (2023). The utility of cardiopulmonary exercise testing in athletes and physically active individuals with or without persistent symptoms after COVID-19. Front. Med. Lausanne.

[22] Komici K., Bencivenga L., Rengo G., Bianco A., Guerra G. (2023). Ventilatory efficiency in post-COVID-19 athletes. Physiol. Rep..

[23] Alsunaidi S.J., Almuhaideb A.M., Ibrahim N.M., Shaikh F.S., Alqudaihi K.S., Alhaidari F.A., Khan I.U., Aslam N., Alshahrani M.S. (2021). Applications of Big Data Analytics to Control COVID-19 Pandemic. Sensors.

